# Indirect evidence for the volume-outcome relationship for corrective surgery for anorectal malformations using the IQWIG V24-07 rapid report methodology

**DOI:** 10.1007/s00383-026-06519-y

**Published:** 2026-07-29

**Authors:** Ophelia Aubert, Timur H. Kuru, Max Samans, Thomas Boemers, Eberhard Schmiedeke, Michael Boettcher, Ekkehart Jenetzky, Ulrich Klaus Fetzner, Carlos Reck-Burneo, Jochen Hubertus, Valeria Solari, Wolfgang Rösch, Miriam Wilms

**Affiliations:** 1 University Hospital Mannheim, Department of Pediatric Surgery, Heidelberg University, Mannheim, Germany; 2 Faculty of Health Sciences Brandenburg, Department of Urology, Brandenburg Medical School Theodor Fontane, Brandenburg an der Havel, Germany; 3 CUROS Urological Centre, Clinic Links vom Rhein, Cologne, Germany; 4 Department of General-, Visceral-, Thorax and Pediatric Surgery, University Hospital Düsseldorf, Medical Faculty of the Heinrich-Heine University Düsseldorf, Düsseldorf, Germany; 5 Clinic for Pediatric and Adolescent Surgery and Urology, Children’s Hospital Cologne, Cologne, Germany; 6 Department of Pediatric Surgery and Urology, Centre for Child and Youth Health, Klinikum Bremen-Mitte, Bremen, Germany; 7 Department of Pediatric and Adolescent Psychiatry and Psychotherapy, University Medical Centre, Johannes Gutenberg University, Mainz, Germany; 8 Faculty of Health/School of Medicine, Witten/Herdecke University, Witten, Germany; 9 Department of General, Visceral and Minimal-Invasive Oncological Surgery, Klinikum Altmuehlfranken, Weißenburg, Germany; 10 Department of Pediatrics, Pediatric Surgery, Brandenburg Medical School, University Hospital Brandenburg an der Havel, Brandenburg an der Havel, Germany; 11 Department of Pediatric Surgery, Marien Hospital Witten, Ruhr-University Bochum, Bochum, Germany; 12 Department of Pediatric and Adolescent Surgery, Kepler University Hospital, Linz, Austria; 13 Department of Pediatric Urology, Marien Hospital St. Elisabeth, Neuwied, Germany; 14 Patient Organization for People with Anorectal Malformations and Hirschsprung Disease (SoMA e.V.), Munich, Germany

**Keywords:** volume-outcome, quality of care, anorectal malformation, formal consensus, rare disease research

## Abstract

**Purpose:**

Anorectal malformations (ARM) are rare congenital anomalies requiring complex surgical correction. Due to low caseload and heterogeneous cohorts, prior studies failed to establish whether higher hospital caseloads improve outcomes. This study applies the IQWiG V24-07 framework to evaluate whether indirect evidence from other surgical procedures can inform a potential volume-outcome relationship in ARM surgery.

**Methods:**

An interdisciplinary expert panel conducted a formal consensus using three steps: (1) identifying suitable source populations; (2) systematically comparing these with the ARM population using 12 IQWiG criteria; and (3) evaluating transferability through structured discussion and voting.

**Results:**

Adult rectal resection was unanimously selected as evidence source population due to robust evidence of a volume-outcome relationship and relevant surgical commonalities, including deep pelvic dissection. Of the 12 transferability criteria, consensus was reached in six. Differences in comorbidities, concomitant treatment, and setting were judged irrelevant for transferability. Differences in sociodemographic and disease-specific characteristics were considered estimable in their impact. Divergent ratings were observed for diagnosis, intervention, endpoints, specialization, and follow-up care. Short-term technical outcomes such as anastomotic leakage were deemed transferable, whereas mortality was considered non-informative in ARM. Functional outcomes required subtype-specific interpretation.

**Conclusion:**

This structured analysis supports the existence of a volume-outcome relationship for ARM surgery.

**Supplementary Information:**

The online version contains supplementary material available at 10.1007/s00383-026-06519-y.

## Introduction

Anorectal malformations (ARM) are complex congenital anomalies with an estimated incidence of 1:2,800 live births [33]. The spectrum of ARM is commonly described using the Krickenbeck classification which distinguishes subtypes according to the anatomy of the malformation [28]. Surgical treatment is demanding and, depending on the specific type of malformation, may range from a single operative intervention to multiple staged corrective procedures. Despite this complexity, previous studies have neither established nor refuted a volume-outcome relationship for complex corrective surgery in ARM, where higher hospital caseload would be associated with better patient outcomes [2, 58]. Those studies have identified several methodological barriers to evaluating such a relationship, including overall low hospital caseload in decentralized health care systems, inhomogeneous patient cohorts, and difficulties in measuring long-term outcomes or associating them to the initial operating centre.

These problems are not confined to ARM but apply to a wide range of other rare and complex diseases. The GRADE working group and other methodological frameworks recommended the use of indirect evidence in situations where direct evidence is unattainable, even under ideal study conditions [16, 23, 39, 40].

The Institute for Quality and Efficiency in Health Care (IQWiG) rapid report V24-07 “Scientific evaluation of the relationship between volume of services and quality of treatment outcome for rare diseases”, outlines criteria for assessing the transferability of indirect evidence concerning the volume-outcome relationship. Specifically, it addresses the transferability of evidence from an evidence source population, where direct evidence of a volume-outcome relationship is available, to a target population comparable to the evidence source population, but which faces the previously described methodological challenges of rare populations.

This study applies the multidimensional methodological approach outlined in the IQWIG V24-07 rapid reports to explore the transferability of the volume-outcome relationship from a common surgical population to corrective surgery of ARM. The aim is to offer the highest achievable level of evidence for the crucial question of whether a volume-outcome relationship exists for the highly complex corrective surgery for ARM in children.

## Methods

The Delphi method was used as a formal consensus method. Consensus was defined as 100% agreement. The authors MW and MS convened an interdisciplinary expert panel comprising two board-certified visceral surgeons (MW, UF), seven board-certified pediatric surgeons (TB, ES, MB, CR, JH, VS, OA), three board-certified urologists (TB, WR, TK) and one board-certified pediatric psychiatrist and methodologist with expertise in rare disease research (EJ). The choice of experts aimed to represent the interdisciplinarity of specialties needed for the treatment of ARM and all experts are clinically or scientifically highly involved in the treatment of ARM. The expert panel was coordinated and moderated by MS.

### Step 1: Selection of the evidence source population(s)

The selection of appropriate evidence source population(s) was discussed by the expert panel. A pre-requisite for inclusion was the availability of strong direct evidence demonstrating a volume-outcome relationship in the proposed source population. In addition, surgical commonalities between the evidence source population and the target population were required. Proposed evidence source population were subjected to open voting by the panel to determine inclusion or exclusion.

### Step 2: Identification of differences between source and target population

Differences between the evidence source population and the target population were identified following the methodology outlined in the IQWiG rapid report V24-07 [31]. For all populations, the criterion “Setting” was analysed with reference to the German healthcare landscape relevant to each population. The characteristics of the evidence source population, adults undergoing rectal resection, were primarily derived from the Evidenced-based Guideline for Colorectal Cancer issued by the German Guideline Program of Oncology, Version 3.01 (June 2025) [14]. Characteristics of the target population, children undergoing corrective surgery for ARMs, were derived from the ERN eUROGEN Guidelines for ARM, Part I-IV [5–8]. Wherever predefined categories could not be sufficiently addressed using the aforementioned source, the most recent and highest-quality research articles, assessed according to GRADE criteria, were used. The summary of the identified differences was made available to the panel in writing.

### Step 3: Structured group discussion and voting

A structured group discussion was conducted among the panel members and moderated and documented by MS. Prior to the discussion, all authors confirmed they understand the identified similarities and differences between the evidence source population and target populations, including aspects outside their primary specialty areas. When a specific aspect was relevant to multiple criteria, it was addressed under the most appropriate criterion only. For example, although “age” is relevant to both Criterion 2 (“Sociodemographic Patient Characteristics”) and Criterion 3 (“Disease-Specific Patient Characteristics”), it was discussed exclusively under Criterion 2, as indicated in Table 1.

During voting on the criteria “sociodemographic patient characteristics”, “disease-specific patient characteristics”, “endpoints”, “specialization and experience” and “setting” MB and TB were not present and therefore did not participate in the vote.

Voting on the transferability of the volume-outcome relationship from the evidence source population to the target population was conducted openly, and dissenting views were documented along with corresponding comments. Transferability was assessed using the predefined categories outlined in the IQWiG rapid report V24-07:

A. The differences between the populations are relevant, and the volume–outcome relationship cannot be applied to the target population.
B. The differences between the populations are relevant but can be minimized to a negligible level by further narrowing the evidence source population and/or refining the intervention criteria.
C. The differences between the populations are relevant when considering the entire target population; however, there is a subpopulation for which these differences are not significant.
D. The differences between the populations are relevant and cannot be eliminated, but their impact on the volume–outcome relationship can be estimated.
E. The differences between the populations do not affect the applicability of the volume–outcome relationship to the target population.

**Fig. 1:**
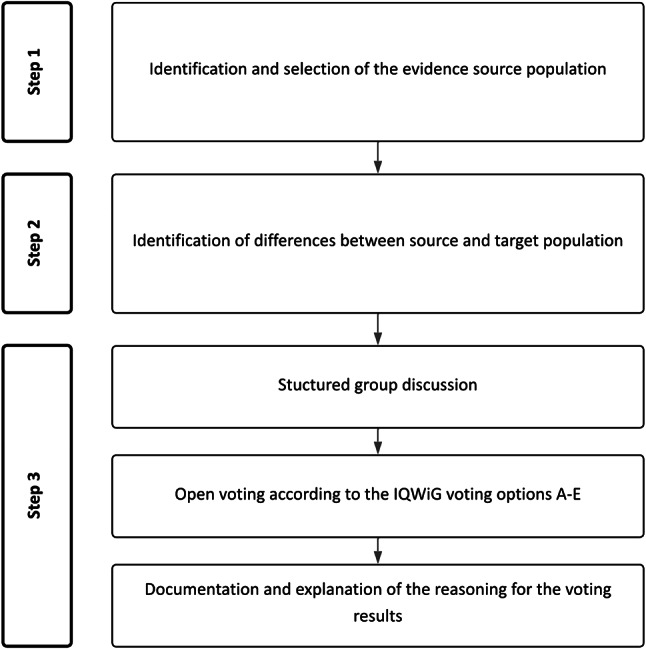
Details of the Delphi process used

The framework from the IQWiG rapid report V24-07 was translated from German into English by the authors, since no official English version was available at the time of this study (Fig. 1).

## Results

### Step 1: Selection of the evidence source population

During the panel discussion, the following candidate evidence source populations were proposed by the panel to cover the spectrum of different types of ARMs:

- Rectal resection in adults, applicable to all patients with ARM.
- Prostatectomy in adults, applicable to male patients with ARM.
- Vaginoplasty in the context of gender-affirmation surgery, applicable to female ARM patients with cloacal malformations.

For rectal resection in adults, a volume-outcome relationship has been demonstrated in numerous studies, with the available evidence summarized in a Cochrane review by Archampong et al. and in the IQWIG V24-02 rapid report [4, 30]. The panel unanimously agreed that this population was suitable as an evidence source population. This decision was based on perceived commonalities in outcome measures as well as comparable key surgical steps. Furthermore, the panel agreed that rectal resection best reflects the surgical core elements shared by all ARM subtypes, as all are characterised by a mislocated anal opening requiring rectal surgery, mobilization of the rectum within the pelvis, and deep rectal anastomosis.

For radical prostatectomy in adults, a volume-outcome relationship has been shown in multiple studies, encompassing both early postoperative outcomes and long-term outcomes, including functional outcomes [54, 60]. The panel considered this population as a potential evidence source to reflect the technical challenges and risks associated with fistula dissection in male ARM subtypes with urethral or bladder neck fistula. However, the panel ultimately decided against its inclusion. This decision was based on the fundamentally ablative nature of prostatectomy, which involves removal of the prostate and seminal vesicles, creation of a vesicourethral anastomosis, and tumour-size-dependent resection of the neurovascular bundle, all factors that substantially limit transferability to the target population.

Vaginoplasty was unanimously excluded due to the absence of direct evidence demonstrating a volume-outcome relationship for this operation in the proposed evidence source population. In addition, the panel considered the inclusion of a separate evidence source population for cloacal malformations unnecessary given the rarity of this ARM subtype, which accounts for approximately 3.5% of all ARM cases [58].

### Step 2: Literature review on the differences between adult rectal surgery and corrective surgery for ARM

The differences between adult rectal surgery and corrective surgery for ARM in children, identified through a structured literature review and according to the 12 criteria of the IQWiG rapid report, are detailed in Supplement A.

### Step 3: Assessment of the transferability of the volume-outcome relationship

Consensus was reached for 6 of the 12 criteria (Table 1). For the domain “comorbidities”, “concomitant treatment” and “setting”, the panel unanimously considered the differences between the evidence source population and the target population to be irrelevant for the transferability of the volume-outcome relationship (rated E). For the criteria “characteristics and course of the disease”, “sociodemographic patient characteristics”, and “disease-specific patient characteristics”, differences were considered to be relevant but estimable in their impact on the volume-outcome relationship, (rated D).

**Table 1:** Summary of the identified differences, voting results and key points of the panel discussion regarding the applicability of differences between adult RC surgery for and corrective surgery for ARM in children across the 12 criteria of the IQWiG rapid report V24-07

Criteria	Rating of experts	Results of panel discussion
1. Characteristics and Course of Disease: - Etiology - Clinical manifestations - Course prior to intervention - Expected course without intervention - Factors influencing course	12x D	Reasoning for rating D: Corrective surgery for ARM in children is usually performed during a sensitive developmental period in infancy. The panel expresses divergent views on whether “early” corrective surgery, within the first three months of life, leads to better functional outcomes compared with later correction. One argument in favour of early correction is the greater neuroplasticity in younger infants, which may facilitate continence. In contrast to adults with RC, who usually have an established continence mechanism prior to disease onset, children born with ARM have not yet developed the cognitive skills required to achieve continence, nor the complex interplay of different functional and anatomical components necessary for defecation. Associated sacral anomalies can affect the function of the sacral nerve plexus, and avoidance of iatrogenic injury to this structure is critical. As neuroplasticity and concomitant sacral anomalies may influence some outcomes, the panel concluded that these differences affect the transferability of the volume-outcome relationship. However, their impact was considered estimable with respect to specific outcomes.

2. Sociodemographic Patient Characteristics: - Age, developmental stage - Sex - Height, weight, BMI - Ethnicity - Geography - Socioeconomic status - Lifestyle factors	10x D	Reasoning for D: Sociodemographic characteristics differ substantially between RC and ARM, most notably with respect to age, body sizes and the relevance of lifestyle or socioeconomic factors. However, the panel considered that these differences are likely to affect outcomes in a similar way across populations, allowing the impact on the volume-outcome relationship to be estimated. Procedural difficulty was judged to be comparable despite differences in body sizes, owing to opposing anatomical constraints, such as small pelvis in infants vs. greater surgical access depth encountered in adults.

3. Disease-Specific Patient Characteristics: - Severity/stage - Prognostic/treatment-relevant factors - Symptom differences by age/maturity - Duration prior to intervention - Biomarkers - Genetic characteristics	10x D	Reasoning for D: Disease-specific characteristics differ between RC and ARM, particularly regarding prognosis, mortality, and the nature of relevant comorbidities. In RC, outcomes are mainly influenced by tumor stage, metastatic burden and acquired comorbidities (e.g. diabetes, renal insufficiency), whereas in ARM they depend largely on the complexity of the malformation and the presence of associated congenital anomalies. Although these factors differ fundamentally between the two populations, the panel agreed that comorbid conditions on both settings substantially influence morbidity and outcomes in a predictable manner, thereby allowing their impact on the volume-outcome relationship to be estimated.

4. Diagnosis: - Differences in procedure or criteria - Qualifications/ experience of diagnosticians	2x E 10x D	Reasoning for D: The initial diagnosis of ARM is generally straightforward, as it relies on inspection of the perineum revealing an absent or abnormally located anal opening. However, due to its rarity, it can be missed during the first neonatal examination, resulting in delayed diagnosis or complications such as intestinal perforation. Accurate classification of ARM subtype is more challenging but essential for correct surgical planning and correction. This requires specialized clinical and radiological expertise to identify the presence and location of the fistula and associated malformations of the urogenital tract. Misdiagnosed of the ARM type may adversely affect, surgical outcome. In conclusion the threshold for achieving a volume-outcome relationship in corrective ARM surgery may be higher than in RC. Reasoning for E: Diagnosing RC and ARM are equally complex and can be optimized by an interdisciplinary process. From this perspective, the panel judged that diagnostic differences do not meaningfully affect the transferability of the volume-outcome relationship. In both populations, the accuracy and quality of correct diagnosis were acknowledged to influence surgical outcomes, but not in a manner that precludes transferability.

5. Comorbidities: - Type/frequency/severity - Effect on outcomes	12x E	Reasoning for E: Comorbidities are common in both populations and influence surgical outcome in each. Although the specific comorbidities differ between RC and AMR, their prevalence regarding the affected organ system is considered comparable. This underscored the importance of interdisciplinary perioperative management in both settings, whether for corrective surgery in ARM or oncological resection in RC.

6. Intervention: - Complexity - Duration of use - Materials used	5x D 7x E	Reasoning for D: The level of the anastomosis differs between RC and ARM surgery. In RC, the anastomosis usually lies in the mid or lower rectum; consequently, anastomotic insufficiency typically leads to abscesses in the pelvis or peritonitis. In contrast, corrective ARM surgery involves a recto-cutaneous, and anastomotic insufficiency usually presents as a perineal wound infection. However, insufficiency of sutures at the urethra or bladder can still present as pelvic abscess or peritonitis. In addition, tension on the mobilized rectum in ARM can increase the risk for pelvic abscess, rectal necrosis, and anal stenosis. This may occur, for example, when a previously created enterostomy is placed too close to the rectum, necessitating taken down and recreation of the enterostomy using a more proximal bowel segment. These differences were considered relevant but estimable in their impact on the volume-outcome relationship. Reasoning for rating E: Despite these differences, the overall technical challenges of the intervention were considered comparable to those encountered in RC surgery. In particular, tumors extending beyond the rectal wall require dissection in close proximity to the surrounding structures, which is analogous to the shared wall between the rectum and urethra or rectum and vagina in ARM patients. From this perspective, the panel judged that these similarities support transferability of the volume-outcome relationship.

7. Implementation of Intervention: - Technology - Stage of development - Duration - Availability - Organ system differences	5x D 7x E	Reasoning for rating D: Since 1982, the team led by Peña introduced and disseminated the PSARP procedure worldwide through a structured teaching model, including formal courses, international surgical mentorship, operative proctoring (including visits to Germany), and dedicated fellowship programs. Despite this standardized teaching mode, the rarity of the disease and the decentralized health care structure have resulted in many pediatric surgeons acquiring proficiency through largely autodidactic learning. This may influence the transferability of the volume-outcome relationship from RC to ARM, as unsupervised or self-directed learning is likely to require a greater number of cases to achieve proficiency compared with structured, supervised training. In addition, the heterogeneity of ARM types, further increasing implementation complexity relative to the largely uniform techniques used in RC. Reasoning for rating E: Conversely, the panel also considered that differences in implementation do not meaningfully affect the volume-outcome relationship, as both populations faced substantial challenges during method adoption. Corrective ARM surgery was introduced globally through standardized teaching, yet, consistently low caseload limited the attainment of high proficiency in many centers. In contrast, RC was initially not standardized, but higher hospital caseloads facilitated learning through favorable training environments.

8. Follow-up Care: - Intensity - Patient adherence - Qualification of professionals	9x D 3x E	Reasoning for rating D: In both populations, the quality of follow-up care influences long-term outcome and may affect the volume-outcome relationship in different ways. However, its impact on transferability is considered outcome specific and dependent on the ARM type. The panel judged that differences in follow-up care are relevant but estimable in their impact on the volume-outcome relationship. Reasoning for rating E: The quality of the follow-up care influences long-term outcomes in both populations. For RC early detection of recurrence is improved through structured follow up care. Whereas in ARM it supports optimization of functional outcomes. Since follow-up care equally affects outcomes in both populations, the panel concluded that these differences do not fundamentally affect the transferability of the volume-outcome relationship.

9. Concomitant Treatments: - Standard use - Necessity - Availability - Prior treatment with same intervention	11x E 1x no vote	Reasoning for rating E: The quality of concomitant treatments and perioperative management likely influences the outcome of corrective surgery, and was considered similarly complex and impactful in both populations. The panel noted that the colostomy formation in children with ARM that often necessary prior to corrective surgery, is associated with a high rate of complications. In particular, if a colostomy is placed too distally in the colon, close to the rectum, the subsequent rectal mobilization and tension free anastomosis of the neoanus might be compromised, necessitating colostomy revision. This can negatively impact the outcome of corrective surgery for ARM. The panel concluded that these differences do no limit the transferability of the volume-outcome relationship.

10. Endpoints: - Expected outcomes - Event rates - Side effects/complications - Timing - Qualifications of assessors	Fecal incontinence: 7x D, 3x C Mortality: 8x A, 1x D, 1x C Anastomotic leak: 10x E Urethral stricture/urethral injury: 10x C Bladder dysfunction: 4x D, 6x C	*Fecal incontinence* Reasoning for rating C: Transferability is limited to ARM subpopulations in whom continence is anatomically achievable. In these patients, surgical expertise is more likely to translate into meaningful functional outcomes. Reasoning for rating D: Fecal incontinence differs fundamentally between RC and ARM. RC patients usually have normal preoperative continence, whereas ARM patients generally have not achieved continence prior to corrective surgery and may lack the anatomical prerequisites due to sphincter or sacral anomalies. Nevertheless, quality of surgery such as nerve preservation, correct positioning of the neoanus, and avoidance of ischemic or infectious complications, significantly influences outcomes in ARM, allowing the impact of on the volume-outcome relationship to be estimated. *Mortality* Reasoning for rating A: Mortality is relevant in RC but is rare following corrective ARM surgery and is mainly driven by associated anomalies rather than surgical performance. Therefore, mortality-based volume-outcome relationships are not transferable. Reasoning for rating C: In high-risk subpopulation of patient with ARM, particularly those with severe associated or syndromic conditions, hospital or surgeon-level factors may influence perioperative mortality. Transferability is therefore limited to these specific subgroups. Reasoning for rating D: Although overall mortality after corrective ARM surgery is low, the influence of comorbidities and hospital-based complication management can be evaluated. Thus, the impact of mortality on the transferability of the volume-outcome relationship can be estimated. Reasoning for rating E: Anastomotic leakage is a well-defined early postoperative endpoint in both RC and ARM and reflects surgical technique and perioperative management. Differences in anatomy and surgery do not limit transferability of the volume-outcome relationship. *Urethral stricture/urethral injury* Reasoning for rating C: This endpoint is specific to ARM subtypes with urogenital involvement and may be associated with technical errors of fistula management such as absence of intraoperative cystoscopy or inadequate bladder catheterization. *Bladder dysfunction* Reasoning for rating C: Bladder dysfunction is often pre-existing and dependent on ARM subtype and associated comorbidities (e.g. spinal malformations), whereas in RC it is typically surgery-induced. Preoperative assessment of bladder function may help in distinguish surgery-related effects from congenital dysfunction. Transferability is therefore limited to ARM subtypes with complex urogenital involvement. Reasoning for rating D: Bladder dysfunction is influenced by major cofactors in both populations that are at least partly independent on the surgical procedure (e.g. (neo)adjuvant therapy and nerve injury in RC vs. associated congenital or spinal anomalies in ARM). Because these cofactors can be documented and adjusted for, their impact on the volume-outcome relationship can be estimated.

11. Specialization and Experience: - Qualifications, specialization, experience	7x E 3x D	Reasoning for rating D: Differences in individual surgical specialization between RC and ARM were considered relevant. Structured subspecialty training exists in adult surgery (e.g. colorectal fellowships), whereas comparable subspecialization in pediatric colorectal surgery is limited. The decentralized nature of pediatric surgical care often results in largely autodidactic learning. Consequently, expertise in ARM is difficult to acquire and maintain proficiency. Given, the technical complexity and rarity of ARM (similar to other pediatric colorectal diseases such as Hirschsprung disease), individual surgeon specialization was considered to be at least as important as in RC surgery. From this perspective, differences in formal training structures were not deemed to preclude transferability of the volume-outcome relationship Reasoning for rating E: The panel emphasized that surgical expertise depends on both disease-specific skills and sufficient procedural volume to maintain proficiency. Given technical complexity and rarity of ARM, individual surgeon specialization was considered to be at least in RC surgery.

12. Setting - Healthcare context - Technology/resources	10x E	Reasoning for rating E: Both RC and ARM surgeries are delivered in an inpatient hospital setting with comparable perioperative resourced and multidisciplinary structures. Corrective ARM surgery is performed almost exclusively in level-1- perinatal centers, with only few exceptions. Certification frameworks, such as those established by ERN eUROGEN/ARM-Net and German Cancer Society (OnkoZert) were considered hospital-level measures to concentrate and benchmark expertise, even though their degree of enforcement differs.

References are provided in Supplement A

A. The differences between the populations are relevant. An applicability of the volume outcome relationship to the target population is not possible

B. The differences between the populations are relevant but can be reduced to a negligible level through further restrictions on the initial population and/or the intervention

C. The differences between the populations are relevant if a transfer to the entire target population is intended. However, there is a subpopulation for which the differences are not relevant

D. The differences between the populations are relevant and cannot be addressed through further restrictions regarding the initial population and/or intervention. However, it is possible to estimate how these differences influence the volume- outcome relationship

E. The differences between the populations have no relevant impact on the applicability of the volume-outcome relationship to the target population

Disagreement was recorded for the remaining six criteria: “diagnosis”, “intervention”, “implementation of intervention”, “follow-up care”, “endpoints”, and “specialization and experience”. For “diagnosis” voting ranged from D to E, reflecting differing views on whether diagnostic complexity affects transferability. Although ARMs are typically recognized on initial inspection, accurate classification of the specific type, which is essential for planning definitive surgery, requires specialized expertise. All panellists agreed that correctly diagnosing the type of ARM, fistula location, and associated anomalies influences outcomes. Some panellists considered the risk of misdiagnosis to increase the threshold for a volume-outcome relationship (rated D), whereas others regarded diagnostic complexity as comparable to that in adult rectal resection (rated E).

For “endpoints”, outcomes were discussed individually and showed the greatest heterogeneity in voting. Anastomotic leak was unanimously rated E, as it directly reflects surgical technique and perioperative management in both populations. Mortality was predominantly rated A, given its rarity following corrective ARM surgery and its strong dependence on associated anomalies rather than surgical quality; minority votes reflected possible relevance in selected high-risk subpopulations. Functional outcomes showed limited or estimable transferability (C or D). Voting on faecal incontinence (rated C and D) reflected fundamental differences in baseline continence and anatomical potential between populations. The panel acknowledged that, in a subgroup of ARM patients with favourable anatomical prerequisites, the outcome “faecal continence” may be a meaningful endpoint for assessing volume-outcome effects. Urethral stricture or injury was rated C, as it is specific to ARM subtypes with urogenital involvement (e.g. recto-prostatic fistula). Bladder dysfunction received C and D ratings, reflecting its frequent occurrence in ARM due to congenital or spinal anomalies, in contrast to adult rectal resection, where it is typically acquired (e.g. pelvic nerve injury, radiotherapy). The panel noted that if documentation of baseline bladder function in ARM patients was documented preoperatively, the impact on the volume-outcome relationship may be more easily assessable.

Concerning “specialization and experience”, the panel highlighted that the decentralized organization of pediatric surgical care, combined with the lack of high-volume centres, often results in self-taught learning for teams performing corrective ARM surgery. Whether this structural context meaningfully affects the transferability of the volume-outcome relationship remained disputed, with ratings ranging from E (no impact on transferability) to D (increased complexity in ARM). Furthermore, in contrast to adult colorectal surgery, pediatric colorectal surgery lacks structured and easily accessible training programs in Germany.

For “intervention” and “implementation of the intervention”, ratings ranged from D to E. Dissent reflected differences in anatomical complexity, learning curves and heterogeneity of disease subtypes in ARM, which may increase technical complexity and learning requirements, particularly in low-caseload settings. However, the majority view was that comparable technical challenges and implementation barriers exist in adult rectal surgery and do not substantially limit the transferability of the volume-outcome relationship.

In summary, based on the IQWiG V24-07 methodology, the panel found that the volume–outcome relationship established in adult rectal cancer surgery is transferable to corrective surgery for ARM in children.

## Discussion

Research on the volume-outcome relationship for ARM is constrained by a structural paradox: decentralized care structures and low hospital caseloads limit the feasibility of adequately powered primary studies, while efforts to centralize care often struggle to gain acceptance in the absence of supporting evidence. In this context, indirect evidence and formal consensus methods to evaluate the use of indirect evidence have become increasingly important in rare disease research and are now widely accepted in the development of clinical guidelines [6, 21, 36, 56].

As ARM comprise a spectrum of different malformations involving the anus, the rectum and the urogenital tract, the panel considered several potential evidence source populations to reflect this diversity. Consensus was reached that rectal resections in adults represent the most appropriate evidence source population for analysing the transferability of the volume-outcome relationships. Adult prostatectomy was considered insufficiently comparable, and limited direct evidence available in other populations, such as in patients receiving vaginoplasty in gender-affirmation surgery, was judged inadequate to support the transferability of a volume-outcome relationship.

For rectal resections in adults, the panel identified relevant similarities between the populations, particularly regarding surgical complexity, pelvic anatomy, the need for meticulous dissection in close proximity to neurovascular structures and adjacent urogenital organs, as well as the reliance on multidisciplinary perioperative care. Although relevant differences between populations were acknowledged, these were largely considered estimable in their impact or unlikely to preclude transferability of the volume-outcome effect.

The choice of endpoints was a key determinant for assessment of transferability. In adult rectal resections, volume-outcome relationships have been most robustly demonstrated for short-term technical outcomes, such as anastomotic leakage, which are closely linked to surgical technique and institutional processes. For ARM, this was considered directly comparable and most suitable for assessing the transferability of the volume-outcome relationship. In contrast, while mortality is a relevant endpoint in adult rectal resections, it was judged largely non-relevant for ARM, due to very low event rates, which are primarily driven by associated anomalies. Functional outcomes such as faecal continence and bladder dysfunction are central in both populations but differ fundamentally in their origins. Thus, these endpoints require subtype- and risk-stratified interpretation, rather than direct transfer of insights from the adult population.

Institutional caseload reflects not only surgeon experience but also organizational expertise, structured perioperative processes, continuous team training and the availability of specialized support services. This is particularly relevant for ARM, where perioperative decision-making, complication management and long-term follow-up depend on coordinated multidisciplinary expertise.

Despite the comparability of the complexity of the treatment between the baseline and the target population explored in this study, many key competencies in ARM surgery are highly procedure-specific, including identification and management of fistulas, preservation of continence mechanisms and treatment of associated urogenital anomalies. These tasks require pediatric-specific surgical expertise that cannot be substituted by experience in adult colorectal surgery alone. Beyond individual surgeon expertise, quality improvement is therefore expected to derive primarily from the transfer of structural principles such as institutional volume, team-based expertise and standardized care pathways. Accordingly, the results of our analysis support not only the existence of a transferable volume-outcome relationship but also the need for specialized pediatric surgical expertise embedded within structured care environments.

In Germany, acquisition of such expertise is further impeded by a decentralized care structure and the absence of high-volume ARM centers. As demonstrated in previous national analyses, caseloads are often extremely low, and experience is frequently acquired in an unsupervised or autodidactic manner [58]. Low annual caseloads are also likely to prolong the acquisition of skills and increase inter-institutional variability in outcomes.

Structured training curricula, mentoring programs, international collaboration, centralized registries and dedicated long-term follow-up pathways are needed in order to improve ARM care. However, such initiatives are difficult to establish and sustain in settings with very low annual caseloads. Without sufficient case concentration, even well-designed educational and clinical frameworks are unlikely to reach their full potential. Furthermore, even with the utmost dedication and professional expertise from local staff, the implementation of centres is likely to fail due to the financial burden of maintaining the recommended multidisciplinary team and needed resources on a cost-effective and permanent basis [8, 59]. Although exact minimum caseload threshold cannot yet be defined, the present findings support the principle that sustained institutional volume is a prerequisite for quality improvement in corrective ARM surgery and overall management of ARM patients.

A limitation of this work lies in the inherent reliance on expert judgment within the methodological framework of the IQWiG. However, in the context of rare diseases and technically complex surgical procedures, where direct evidence is often unattainable, even under ideal study conditions, informed expert assessment remains indispensable.

This structured evaluation demonstrated that ARM constitutes a rare and heterogeneous disease entity with unique pediatric surgical challenges. The fundamental mechanisms that explain improved outcomes with higher institutional volume-experience, team coordination, routine management of complications and structured perioperative care are equally relevant. Lastly, while this analysis was conducted within the German healthcare context, the underlying challenges, low case numbers, decentralized care and limited structured subspecialization are shared by many healthcare systems. The findings are likely to be applicable to other countries with comparable pediatric surgical care structures particularly within the ERN eUROGEN.

## Conclusion

In summary, based on a formal consensus assessment, this study supports the transferability of a volume-outcome relationship to corrective surgery for ARM and provides a methodological foundation for future clinical and policy discussions on the centralizing of care for children with ARM.

## Supplementary Information

Below is the link to the electronic supplementary material.

Supplementary Material 1

## Data Availability

No datasets were generated or analysed during the current study.
