# Supplementary material for: Indirect evidence for the volume-outcome relationship for corrective surgery for anorectal malformations using the IQWIG V24-07 rapid report methodology
Source: Pediatr Surg Int. 2026 Jul 29;42(1):322. doi: 10.1007/s00383-026-06519-y (PMC13421219; doi:10.1007/s00383-026-06519-y)
Supplement: Supplementary file 1 — Supplementary Material 1 [file 383_2026_6519_MOESM1_ESM.docx]

**Supplementary Material**

**Annex A**

**Framework to evaluate the transferability of the volume-outcome relationship from the evidence source population “rectal cancer resection (RCR)” to the target population “children undergoing corrective surgery for anorectal malformation (ARM)” according to the IQWIG V24-07 rapid report, with a description of population-specific characteristics (RCR, ARM).**

**1. Characteristics and Course of the disease**

- Etiology
- Manifestations of the disease
- Methods of measuring disease manifestations
- Course of the disease prior to intervention
- Expected course without intervention
- Factors influencing disease progression

**RCR**

Rectal cancer (RC) develops through a multifactorial etiology involving both genetic predisposition and environmental factors. Most cases occur sporadically, with approximately 5% attributable to well-defined monogenetic-inherited syndromes such as Lynch syndrome and familial adenomatous polyposis. An additional 20-30% show familial clustering. Environmental and lifestyle factors play a substantial role; tobacco use, excessive alcohol intake, and obesity (BMI >25 kg/m2) are the most relevant. Chronic inflammatory bowel disease increases the risk for RC. Clinical manifestations depend on tumor stage. Early-stage disease is often asymptomatic and is typically diagnosed through screening colonoscopy. Symptoms include hematochezia, iron-deficiency anemia, changes in bowel habits, abdominal pain, intestinal obstruction, and unintentional weight loss. Advanced disease may manifest with palpable abdominal masses, hepatomegaly due to liver metastases, or other signs of metastatic spread [34].

Diagnosis is confirmed by histopathological examination of biopsy samples obtained during colonoscopy. Early detection substantially impacts prognosis. In Germany, the relative 5-year survival rates for RC is approximately 60% [48].

**ARM**

The etiology of ARM remains incompletely understood and is considered multifactorial. Approximately 60% of patients have associated congenital anomalies, most commonly cardiovascular, gastrointestinal, spinal, or limb defects, often part of syndromic constellations such as the VACTERL association.

Clinical presentation depends on the presence or absence of a fistula and varies between sexes. Prenatal detection remains rare. However, diagnosis is usually made at birth by clinical examination revealing an absent or misplaced anal opening (fistula). Additional investigations, including cardiac, renal, and spinal ultrasound, MRI, and voiding cystourethrography, are essential to identify associated anomalies [7]. Classification according to the Krickenbeck classification is challenging and relies on imaging such as high pressure colostogram or MRI [27]. Perineal fistula may occur in both sexes and represents the most common and least complex variant. In males, fistulas can terminate in the bulbar or prostatic urethra, or more rarely in the bladder neck. In females, vestibular fistulas are characterized by an opening of the rectum into the vaginal vestibule. Cloacal malformations are characterized by a single perineal opening where the rectum, vagina, and urinary tract converge in a common channel which can be short (<3 cm) or long (>3 cm). ARM without fistula or those presenting with rectal stenosis are rare variants.

ARM with perineal or vestibular fistulas may allow partial passage of meconium, whereas ARM without an outlet or complex variants often cause progressive intestinal obstruction, abdominal distension, and risk of sepsis if untreated. The overall mortality of ARM is low.

**2. Sociodemographic Patient Characteristics**

Do the baseline population and target population differ in terms of sociodemographic characteristics of the patients? The following features may be especially relevant:

- Age and developmental stage
- Sex
- Height, weight, body mass index (BMI)
- Ethnicity
- Geographical factors
- Socioeconomic status
- Lifestyle factors such as smoking or physical activity

**RCR**

RCR is performed almost exclusively in adults, with a balanced sex distribution [48]. RC in children is exceedingly rare, with an estimated incidence of 15 new cases per year in Germany [9]. Major risk factors include age, family history (including inherited syndromes such as Lynch syndrome and familial adenomatous polyposis), obesity (BMI >25 kg/m2), smoking, alcohol consumption, and a chronic inflammatory bowel disease [34].

**ARM**

ARM is typically detected shortly after birth. Prenatal detection remains an exception. ARM with fistula typically allow partial passage of meconium and may occasionally be overlooked, particularly perineal fistulas, resulting in delayed diagnosis [21]. ARM show a slight male predominance overall [23]. No clear ethnic or geographic differences have been identified. Lifestyle and environmental risk factors are unknown. Socioeconomic factors may influence long-term functional outcomes through access to specialized care [26, 45]. Regular follow-up in dedicated pediatric colorectal centers and structured bowel management programs improve continence and quality of life, highlighting the importance of multidisciplinary care rather than demographic determinants [5, 6].

**3. Disease-Specific Patient Characteristics**

Do the baseline population and target population differ in terms of disease-specific patient characteristics? The following characteristics may be of particular importance:

- Severity or stage of the disease
- Prognostic or other treatment-relevant factors that may influence the relationship between volume and outcome
- Age at disease onset
- Age- or maturity-related differences in symptomatology
- Duration of the disease prior to intervention
- Biomarkers reflecting severity or progression of the disease
- Genetic characteristics

**“Age-related” aspects are addressed under Criterion 2.**

**RCR**

At diagnosis, approximately one-quarter of patients present with distant metastases. About 10% undergo surgery with palliative intent. Despite advances in treatment, relative 5-year survival rates remain around 60%. Neoadjuvant chemoradiotherapies, may substantially improve overall survival depending on the stage of the disease according to the UICC classification. Carcinoembryonic Antigen (CEA) is elevated in approximately 30% of cases and is used to monitor disease progression. Molecular markers such as K-RAS and BRAF mutations are of major prognostic and therapeutic relevance, supporting an individualized treatment approach [34].

**ARM**

ARM is a benign, non-progressive condition. Prognosis depends on malformation type and associated anomalies. Sacral and spinal anomalies, sphincter development, and urogenital malformations are key determinants of bowel and urinary function. In cloacal malformations, functional outcomes correlate with anatomical parameters such as common channel length. ARM subtypes vary in their complexity regarding corrective surgery. ARM variants with adequate drainage allow elective correction without a preceding colostomy, while other forms require decompression by colostomy [8]. Definitive reconstructive surgery is usually elective, and delayed reconstruction do not worsen outcomes. Long‑term quality of life can be optimized through structured follow‑up care, bowel management programs, dietary counseling, and ongoing multidisciplinary support.

**4. Diagnosis**

Are there differences in diagnostic procedures between the baseline population and target population?

The following questions should be considered:

- Do the procedures or criteria for diagnosis differ between the baseline and target populations?
- Do the qualifications, specialization, or experience of those involved in diagnosis vary?

**RCR**

RC is diagnosed through histological examination of biopsy samples during colonoscopy or rectoscopy prior to the oncological resection. Colonoscopy in adults is standardized and broadly with structured reporting of both endoscopic findings and corresponding pathology.

Most general pathologists are qualified to diagnose RC on biopsy or resection specimens, however rare subtypes or cases with atypical features may require reference centers reevaluation. Pretherapeutic imaging aims to determine the presence of distant metastases or lymph node metastasis. Staging of RC includes, if possible, a complete colonoscopy, abdominal ultrasound, CEA testing, and a chest X-ray, a rigid rectoscopy, pelvic MRI scan and rectal endosonography. RC is staged using the AJCC/UICC TNM system, which integrates tumor depth (T), nodal status (N), and distant metastasis (M), as well as histologic grade, and margin status. Postoperative assessment further evaluates resection completeness and histologic features, including integrity of the mesorectal fascia and the number of lymph nodes retrieved [34].

**ARM**

Diagnosing begins with clinical examination. The initial assessment includes inspection of the perineum to determine the presence and position of a fistula. Absence of clinically visible fistula combined with failure to pass meconium requires prompt surgical intervention as outlined in paragraph 6. Accurate diagnosis and preoperative assessment require close collaboration among pediatric colorectal, urologic, and radiologic specialists [7]. Reliable interpretation of imaging and endoscopic findings depends on specialized expertise and access to appropriate pediatric instruments and facilities. Unlike oncologic conditions, ARM have no formal TNM or prognostic staging system. The Krickenbeck classification is commonly used to distinguish subtypes. Diagnostic protocols are not standardized, and practices may differ amongst different hospitals and countries. Diagnostic evaluation focuses on defining the precise anatomy to guide reconstruction of the anorectal canal and restoration of sphincter function. Additionally, appropriate investigations should be initiated to identify associated malformations, particularly those involving the spine, heart, and renal tract, or occurring as part of syndromic constellations.

The level of the fistula and its relationship to the urogenital tract can be evaluated using contrast studies (e.g., high-pressure colostogram) [41]. In cloacal malformations, cystoscopy and vaginoscopy should be performed to delineate the anatomy of the urinary and genital tracts and to measure the length of the common channel. In some cases, such as rectal stenosis, MRI investigation is essential to exclude Currarino triad, a condition characterized by a sacral defect, presacral mass, and ARM [1].

**5. Comorbidities**

Do patients in the baseline population or those receiving the baseline intervention differ from those in the target population or those receiving the target intervention in terms of comorbidities?

The following may be particularly relevant:

- Differences in type, frequency, or severity of comorbid conditions between populations
- The extent to which comorbidities may affect treatment outcomes or the effectiveness of the intervention

**RCR**

Syndromic comorbidities and other congenital conditions are rare. Well-defined hereditary colorectal cancer syndromes (e.g. Lynch syndrome, familial adenomatous polyposis, MUTYH-associated polyposis, and hamartomatous polyposis syndromes) make up 5% of all colorectal cancer cases. These hereditary forms may present with extracolonic features (dermatologic, ophthalmic, dental, endocrine, vascular, reproductive). The most common comorbidities among RC patients are age-related systemic diseases including hypertension, diabetes, cardiovascular disease, and chronic kidney disease. Over recent decades, the overall prevalence of comorbidity in this population has increased from 47% to 62%. Multimorbidity is particularly common in elderly patients and those of lower socioeconomic backgrounds. A high comorbidity burden is a strong predictor of adverse outcomes, including increased rates of severe complications and reduced overall survival [34].

**ARM**

Approximately 60 % of patients have associated congenital anomalies [23]. Systematic screening of the renal, cardiac, vertebral, spinal, and genital systems is essential at diagnosis [7]. Urologic anomalies are common and increase with malformation complexity [12, 13, 16]. They include vesicoureteral reflux, hydronephrosis, renal dysplasia, and neurogenic bladder dysfunction. High-grade vesicoureteral reflux occurs in nearly 20% of patients, and even those with normal initial renal screening may later develop urinary tract abnormalities [51]. These conditions contribute to morbidity by predisposing to recurrent urinary tract infections and progressive renal impairment, and warrant lifelong urologic surveillance. Cardiac anomalies occur in 20–40% of patients, with prevalence and severity correlating with ARM complexity [37]. Most are septal defects or other non-cyanotic malformations, but severe lesions such as Tetralogy of Fallot or transposition of the great arteries also occur [23]. Congenital heart disease significantly influences perioperative risk and long-term prognosis. Spinal and sacral abnormalities, including tethered cord, sacral agenesis, and lipomyelomeningocele, are observed in 25–50% of patients and affect bowel and urinary continence [33, 35]. These findings underscore the need for multidisciplinary assessment involving pediatric urology, cardiology, neurosurgery, and orthopedics. Misclassification of ARM may lead to incorrect corrective surgery.

Syndromic associations are common, particularly VACTERL association, Currarino triad, and, less frequently, Down, Townes–Brocks, MURCS, and OEIS syndrome [53]. In isolated ARM, perioperative mortality is extremely low and typically related to severe cardiac or renal anomalies rather than to the anorectal defect itself [32, 46]. After corrective surgery, bowel dysfunction, including constipation, fecal incontinence, and soiling, affects many patients (17–77%), as does urinary incontinence [11, 46].

**6. Intervention**

Do the baseline population and target population differ with regard to the intervention itself?

Relevant aspects may include:

- Complexity of the intervention
- Duration of implementation (how long the intervention has been in use)
- Materials used

If newer procedures have replaced older interventions, it must be assessed which of these procedures are transferable and which are not. If necessary, the inclusion and exclusion criteria of the initial research question must be adjusted so that only the procedure deemed transferable is considered.

**RCR**

RCR is technically demanding and demonstrates a clear volume-outcome relationship at both hospital and surgeon level [4, 30].

For rectal tumors in the upper third of the rectum, partial mesorectal excision (PME) extends 5 cm distal to the tumor margin. For mid and low rectal cancers, total mesorectal excision (TME) is required, with preservation of the hypogastric plexus and autonomic nerves. Low rectal tumors, which involved the anal canal or sphincter may require cylindrical abdominoperineal excision, as well as resection of the levator ani.

Reconstruction after a low anterior resection generally involves a colorectal or coloanal anastomosis. Colon J-pouch or side-to-end configurations offer superior functional outcomes compared to transverse coloplasty. Tension-free, well-perfused anastomoses require splenic flexure mobilization, and a temporary diverting stoma is recommended for low anastomoses after TME.

Intraoperative liver inspection should be performed even when preoperative imaging is negative. Frozen sections should be used selectively when results are expected to change management, for example to assess metastatic spread or resection margins in deep seated rectal tumors.

Most rectal resections are performed in lithotomy position. Transanal total mesorectal excision (TaTME) combines transanal and abdominal approaches. Laparoscopic and robotic resections show comparable outcomes to open surgery if performed by experienced surgeons.

Radical resection for CRC aims to remove the tumor with adequate lymphadenectomy. The extent of the resection is determined by central vascular ligation. For paracolic resections, a bowel margin of 10 cm is generally considered sufficient. Overall, rectal resections follow standardized oncologic principles based on tumor location [34].

**ARM**

Corrective surgery for ARM is technically demanding and requires advanced pediatric colorectal expertise. Procedural complexity depends on the type of malformation, increasing from perineal and vestibular fistulas to rectourethral and bladder-neck fistulas, and peaking in cloacal malformations. Successful outcomes require precise knowledge of pelvic anatomy, ARM subtypes, and accurate preoperative diagnosis [10].

Corrective surgery for ARM can be performed via a perineal access or combined abdominal and perineal approach (open or laparoscopic-assisted), depending on the type and level of the malformation, presence of a fistula, and institutional resources [8]. ARM with visible fistula (e.g. perineal and vestibular) can often be corrected in the neonatal period or within the first few months of life using an established technique such as (posterior sagittal anorectoplasty (PSARP), perineal preserving PSARP (PPP), anterior sagittal anorectoplasty (ASARP), and a single-stage repair may be appropriate. Malformations without a clinically visual fistula usually require initial diversion with a colostomy followed by definitive repair.

The central principle of ARM repair is the correct placement of the neoanus within the sphincter complex to preserve continence. Intraoperative electrical stimulation is routinely used to identify the center of the sphincter and guide accurate neoanal positioning. Dissection must remain close to the rectal wall to protect the sphincter and avoid injury to adjacent structures, particularly the urinary tract and vagina. This is particularly delicate in females, where the rectum and vagina are separated by a thin common wall prone to perforation. In cloacal malformations, complexity increases as the rectum, vagina, and urinary tract form a common channel. In males, precise identification and ligation of the rectourethral or bladder-neck fistula are essential to prevent complications such as a remnant of the original fistula (ROOF) [44].

Surgical exposure is typically obtained in the lithotomy position for less complex repairs (e.g. ASARP) and in prone position for classic PSARP and cloacal reconstructions. Laparoscopic-assisted techniques require full-body sterile preparation. The lithotomy position offers optimal access but carries risks of nerve compression, deep vein thrombosis, and pressure injuries during longer procedures, whereas the prone position offers superior perineal exposure. The optimal timing of surgery remains debated; current evidence shows no clear benefit of neonatal versus delayed repair for perineal or vestibular ARM [31, 42]. Surgical timing should be individualized based on patient condition, comorbidities, and surgical expertise.

Essential surgical equipment includes pediatric laparoscopic, abdominal and transanal instruments, electrocautery and vessel-sealing devices; a Lone Star® retractor system for perineal exposure, fine suture materials for hand-sewn anastomosis; and an electrical stimulation device for sphincter mapping. Perioperative antibiotic prophylaxis is standard, and many institutions administer multi-day antibiotic coverage [49].

Overall, high-quality management of patients with ARM requires meticulous multidisciplinary perioperative planning, specialized pediatric surgical expertise, and structured long term follow-up protocols.

**7. Implementation of the Intervention**

Are there differences in how the intervention is carried out between the two populations?

Relevant aspects may include:

- Technology used
- Stage of technological development
- Duration of treatment
- Availability of technologies or resources
- Treated organ or organ system – possibly with differences due to growth or maturation processes that may facilitate or complicate the intervention

**RCR**

Surgical resections for RC started in the late 19th century and became standardized by the mid-20th century. TME, developed in the early 1980s, is now the standard approach for mid and low rectal cancer. Prior to TME, incomplete mesorectal excision was associated with local recurrence rates above 20%. After the introduction of TME, these rates decreased to below 10%, largely independent of adjuvant therapy. Laparoscopic RC surgery was introduced in the 1990s, with the COLOR II trial demonstrating oncological outcomes equivalent to open surgery and improved short-term postoperative outcomes [55]. In Germany, no formal training curriculum initially existed for laparoscopic rectal resection; nevertheless, the proportion of minimally invasive procedures increased from 12.3% to 48.1% between 2007 and 2016 across 30 certified cancer centers [50].

The transanal total mesorectal excision (TaTME), first described in 2010, represents a further technical evolution. Owing to its complexity and the increased risk of urethral, prostate, and nerve injury, TaTME requires structured training and proctoring programs. Proficiency is generally reported to require at least 40 supervised TaTME procedures [34, 47].

**ARM**

Surgical correction of ARM evolved significantly over the last decades. The introduction of the PSARP by Alberto Peña in 1982 revolutionized ARM surgery by enabling direct visualization of the pelvic floor and precise anatomical reconstruction [14]. Subsequent modifications, such as the ASARP for perineal fistula and the more recent PPP for rectovestibular fistulas, aim to minimize perineal trauma [8, 62]. In 1999, Georgeson introduced the laparoscopic-assisted anorectal pull-through (LAARP), which reduced tissue trauma compared to open approaches [19]. Operative duration varies by complexity: approximately 1.5-2 hours for ASARP, 2-2.5 hours for PSARP or LAARP, and up to 6-8 hours for extensive cloacal reconstructions, with blood loss generally being minimal [52, 61]. Complex cases frequently require simultaneous urologic or gynecologic intervention, and cloacal reconstructions are often managed by multidisciplinary teams [57]. All ARM procedures require pediatric anesthesia, neonatal intensive care support, and advanced perioperative resources, particularly given the high prevalence of associated comorbidities, which are typically available only in specialized pediatric centers. Technological advances have progressively refined ARM reconstruction. Laparoscopic approaches are now standard for many complex malformations, and robotic-assisted surgery is starting to be implemented in selected centers [17].

**8. Follow-up Care**

Are there differences in follow-up care between the baseline and target populations? Particularly relevant aspects may include:

- Intensity of follow-up care
- Expected patient adherence to follow-up protocols
- Qualification of professionals providing follow-up care

**RCR**

In adults undergoing RCR, follow-up care is protocol-based and primarily focuses on oncologic surveillance. For patients with stage I disease (UICC stage I) who have undergone R0 resection, routine follow-up is generally not required because of the low risk of recurrence; however, colonoscopic surveillance is advised in accordance with standard screening guidelines. Patients with stage II and III disease require structured follow-up, as approximately 80% of recurrences occur within the first two years and are uncommon after five years. Expert consensus recommends check-up visits every six months for the first two years, followed by long-term colonoscopy surveillance to detect secondary neoplasms.

Quality of life may be adversely affected by the presence of a stoma, advanced age, or adjuvant therapy. Between 20% and 35% of patients develop treatable psychological disorders. Low anterior resection syndrome (LARS), characterized by bowel or urinary dysfunction, is common after rectal resections. Adherence to RC follow-up programs is generally high, reflecting the presence of standardized care protocols [34].

**ARM**

Lifelong follow-up is recommended for all patients with ARM [6]. Follow-up care should be coordinated by an interdisciplinary colorectal team led by a pediatric surgeon and include pediatric urology, gynecology, gastroenterology, psychology, physiotherapy, nursing, and nutrition [5, 6]. Transition planning should begin in early adolescence, with adult healthcare providers identified before transfer to ensure continuity of care. Follow-up intensity varies by age and clinical stability. During infancy and early childhood, visits are frequent, often every few weeks to months, to monitor postoperative healing, initiate bowel management, and detect urinary complications. As continence stabilizes, intervals can be extended to annual or biannual visits. Functional assessment remains central, addressing bowel, urinary, and sexual outcomes. Long-term functional morbidity remains common, underscoring the need for structured lifelong care. Constipation affects up to 80% of patients, fecal soiling or incontinence 15–75%, and urinary incontinence 10–30%, with higher rates in complex ARM and cloacal malformations [24, 46]. Sexual dysfunction and reduced fertility are reported in both sexes, particularly after complex reconstructions or with associated urogenital anomalies [46]. Psychosocial quality of life is lower compared to healthy peers, with increased anxiety, reduced body confidence, and social challenges, especially in adolescents.

Adherence to follow-up schedules and treatment depends on effective counseling and parental education. Socioeconomic factors may also influence compliance, highlighting the importance of early education and individualized, written care plans [26, 45]. Currently, standardized follow-up guidelines are lacking.

**9. Concomitant Treatments**

Are there differences in pre- or concomitant treatments between the initial and target populations?

Aspects to consider include:

- Standard use of pre- or concomitant treatments
- Necessity of certain pre- or concomitant treatments
- Availability and accessibility of such treatments
- Prior treatment with the same intervention

**RCR**

In adult patients undergoing RCR, concomitant treatments are determined by tumor stage and location. Neoadjuvant treatment is standard for locally advanced rectal cancer (UICC stage II and III). Options include long-course chemoradiotherapy with concurrent fluoropyrimidine-based chemotherapy, short-course radiotherapy (5 × 5 Gy), or total neoadjuvant therapy (TNT), which combines induction or consolidation chemotherapy with radiotherapy prior to surgery. Neoadjuvant treatment aims to downstage the tumor, increase the likelihood of sphincter-preserving surgery, and reduce local recurrence rates. Adjuvant chemotherapy is recommended for patients with stage III disease and selectively for high-risk stage II disease. Standard adjuvant regimens include fluoropyrimidine monotherapy or combination regimens with oxaliplatin (FOLFOX). Perioperative management follows enhanced recovery after surgery (ERAS) protocols, including early mobilization, multimodal analgesia, and structured nutritional support. These concomitant treatments are well standardized, widely available, and integrated into certified cancer center pathways [34].

**ARM**

For ARM, there is no standardized preoperative medical regimen prior to corrective surgery. Preoperative preparation focuses on clinical stabilization (fluid/electrolyte balance, analgesia), assessment of associated anomalies, and timing of repair according to anatomy and center expertise.

A diverting colostomy is indicated in selected cases, most commonly in ARM without fistula, rectourethral or bladder‑neck fistulas, and cloacal malformations, or when decompression and perineal hygiene cannot be maintained safely [8]. Stoma creation should balance adequate decompression with low complication risk. Reversal is performed once the neoanal anastomosis has healed and clinical stabilization. Perioperative care includes antimicrobial prophylaxis, intravenous fluids, and multimodal analgesia. There is no ARM-specific neoadjuvant or adjuvant medical therapy

Postoperative bowel management may be required, in infants with persistent constipation or fecal incontinence. In these cases, laxatives and stool‑softening agents (e.g., polyethylene glycol) or bowel-irrigations may be employed.

Access to specialized pediatric colorectal care can be limited outside tertiary centers. Redo-surgery is uncommon but has been described, especially in cases with persistent soiling and/or constipation [3]. Mislocation of the anus is a potential cause requiring reoperation.

**10. Endpoints**

Are there differences between the endpoints relevant for the target research question and those assessed in studies addressing the initial research question?
Relevant aspects may include:

- Expected treatment outcomes
- Anticipated event rates for event-based endpoints central to the researches for event-based endpoints:
- Expected manifestation of side effects or complications:
  If complications or side effects present differently between populations, attribution of results to the appropriate population may be difficult.
- Timing of endpoint assessment: Differences in the expected timing of complications must be considered when interpreting results This must be taken into account when interpreting results. In particular, longer, or developmentally adjusted follow-up periods may be required for children due to growth and developmental processes compared with adults.
- Qualification, specialization, and experience of individuals assessing the endpoints.

**RCR**

The IQWiG rapid report V24-02 identified a correlation between hospital or surgeon volume and several outcome measures for RCR. Specifically, higher case volumes were associated with lower rates of in-hospital, 30-day, 90-day, and 5-year mortality, as well as reduced failure-to-rescue events, overall complications, renal failure, and permanent stoma formation. Similarly, the Cochrane review by Archampong et al. confirmed a volume–outcome association for 5-year mortality, anastomotic leakage, permanent stoma rates, and preservation of continence [4]. In contrast, the IQWiG report V24-02 found no significant relationship between hospital volume and outcomes such as postoperative respiratory failure, wound infection, disease progression, hospital of stay duration, positive circumferential resection margins, or reintervention rates. Endpoints in adult colorectal surgery are predominantly derived from hospital discharge records or clinical registry records [34].

**ARM**

The “Anorectal malformation and outcome review (ARMOUR)” project systematically evaluated outcome reporting on ARM. Of 118 included studies, 339 distinct outcomes were identified. Sample size ranged from 1 to 1,206, patients, with many studies lacking clear descriptions of surgical techniques (29%) and age categories (21%) [25]. Gastrointestinal and renal and urinary outcomes were reported most frequently [6]. Specifically, the most commonly reported outcomes included postoperative complications, bowel function, bladder function and testicular problems, social outcomes, stoma problems, sexual function and pre- and perioperative complications. Constipation was the most frequently reported gastrointestinal endpoint. Most outcomes were clinician-reported (59%), while only 10% were patient-reported. Measurement tools varied widely, including both validated instruments and study-specific, non-validated measures. The Krickenbeck classification was the most commonly applied rating scale for postoperative results. Many studies lacked clear outcome definitions and did not report validation of their measurement instruments.

**11. Specialization and Experience of Treating Professionals**

Are the qualifications, specialization, and experience of the treating professionals comparable between the initial and target populations and interventions?

**RCR**

In Germany, RCR is performed by surgeons, specialized in general and/or visceral surgery, often with additional sub-specialization or international certifications, such as the European Board of Surgical Qualification in Coloproctology (EBSQ).

Currently, 326 colorectal cancer centers certified by the German Cancer Society exist and each is mandated to have two senior colorectal cancer surgeons involved in every oncological resection performed at the institution. Since 2024, nationwide mandatory minimum caseloads have been implemented, requiring a minimum of 30 oncologic colon and 20 oncologic rectal resections per hospital per year [18, 38]. Approximately 50,000 oncological colorectal resections and an additional 26.000 sigmoid resections for diverticular disease are performed in Germany contributing to surgical experience [34, 43].

**ARM**

Corrective surgery for ARM is performed by pediatric surgeons. In Germany, pediatric surgery covers nearly all childhood surgical conditions except cardiac surgery and neurosurgery. Formal subspecialization exists in areas such as pediatric urology or orthopedics, but not in pediatric colorectal surgery.

Although approximately 50 centers from twenty European countries are certified healthcare providers for ARM treatment within ERN eUROGEN, this voluntary certification has not resulted in effective centralization of care, increased caseload at certified hospitals, or fewer hospitals performing ARM surgery [58]. This is noteworthy, as ARM repair is typically elective and theoretically well suited for centralization.

ARM is rare, with approximately 300 corrective surgeries performed annually in Germany. Other major pediatric colorectal disorders, such as Hirschsprung disease, are even rarer, with around 160 surgeries per year [58]. Given the broad spectrum of ARM subtypes, individual center volumes, particularly for complex anomalies, are low, limiting experience and skill transfer.

Completion of corrective ARM surgery is not required for board certification in pediatric surgery in Germany, and colorectal expertise is typically acquired through post-residency training at specialized centres. No mandatory quality control measures exist for ARM or other congenital anorectal anomalies in Germany. Although ERN eUROGEN guidelines provide recommendations for management and follow-up, many are based on expert consensus due to the rarity and heterogeneity of ARM [6]. Research is constrained by small sample sizes, ARM subtype variability, and frequent associated comorbidities, particularly spinal cord anomalies affecting continence.

**12. Setting**

Are there differences in the setting between the initial and target research questions?
Relevant aspects may include:

- Healthcare context
- Processes used
- Conditions under which the intervention is conducted
- Preparation for the intervention
- Availability of technology and resources

**RCR**

CRC is common, affecting about 1 in 19 women and 1 in 15 men. In Germany, 46,325 oncological CRC resections were performed in 2022 across 1,052 hospitals, with institutional case load ranging from 1 and 241 procedures per site (mean 44, median 35); 212 hospitals performed fewer than 15 annually. The average travel time to a hospital was 11 minutes (approximately 7 km) [28].

Although most hospitals regularly perform colorectal surgery, many remain low-volume centers. To address the established volume-outcome relationship, Germany introduced mandatory minimum caseload requirements for colon and rectal resections in November 2024. Aspects related to “processes used”, “conditions under which the intervention is conducted” and “preoperative preparation” are evaluated under Criteria 3 and 6 [34].

**ARM**

ARM are rare congenital conditions with an incidence of about 1 in 2,800 live births. In Germany, around 2,000 corrective operations for ARM were performed between 2016 and 2021 across 113 hospitals, reflecting substantial decentralization [58].

Although corrective ARM surgery is elective and plannable, care remains fragmented. Surgical preparation involves multidisciplinary assessment, imaging, and perioperative planning [8]. However, availability of specialized pediatric surgical equipment (e.g. pediatric laparoscopic instruments and intraoperative nerve stimulators) and surgical expertise, varies between hospitals, potentially limiting implementation of standardized care pathways and affecting outcomes.

Overall, ARM care in Germany is decentralized and marked by heterogeneous institutional experience, challenging experience consolidation and evaluation of potential volume-outcome relationships.
